# Associations of BNT162b2 vaccination with SARS-CoV-2 infection and hospital admission and death with covid-19 in nursing homes and healthcare workers in Catalonia: prospective cohort study

**DOI:** 10.1136/bmj.n1868

**Published:** 2021-08-18

**Authors:** Carmen Cabezas, Ermengol Coma, Nuria Mora-Fernandez, Xintong Li, Montse Martinez-Marcos, Francesc Fina, Mireia Fabregas, Eduardo Hermosilla, Angel Jover, Juan Carlos Contel, Yolanda Lejardi, Belen Enfedaque, Josep Maria Argimon, Manuel Medina-Peralta, Daniel Prieto-Alhambra

**Affiliations:** 1 Public Health Secretariat, Department of Health, Generalitat de Catalunya, Barcelona, Spain; 2 Direcció assistencial d’Atenció Primària i a la Comunitat, Institut Català de la Salut (ICS), Generalitat de Catalunya, Barcelona, Spain; 3 Centre for Statistics in Medicine, NDORMS, University of Oxford, Oxford, UK; 4 Institut Català de la Salut (ICS), Generalitat de Catalunya, Barcelona, Spain; 5 Idiap Jordi Gol, Universitat Autonoma de Barcelona, Barcelona, Spain; 6 Chronic Care Program, Integrated Health and Social Care Plan, Department of Health, Generalitat de Catalunya, Barcelona, Spain; 7 Generalitat de Catalunya, Barcelona, Spain; 8 Department of Medical Informatics, Erasmus University Medical Center, Rotterdam, Netherlands

## Abstract

**Objective:**

To determine associations of BNT162b2 vaccination with SARS-CoV-2 infection and hospital admission and death with covid-19 among nursing home residents, nursing home staff, and healthcare workers.

**Design:**

Prospective cohort study.

**Setting:**

Nursing homes and linked electronic medical record, test, and mortality data in Catalonia on 27 December 2020.

**Participants:**

28 456 nursing home residents, 26 170 nursing home staff, and 61 791 healthcare workers.

**Main outcome measures:**

Participants were followed until the earliest outcome (confirmed SARS-CoV-2 infection, hospital admission or death with covid-19) or 26 May 2021. Vaccination status was introduced as a time varying exposure, with a 14 day run-in after the first dose. Mixed effects Cox models were fitted to estimate hazard ratios with index month as a fixed effect and adjusted for confounders including sociodemographics, comorbidity, and previous medicine use.

**Results:**

Among the nursing home residents, SARS-CoV-2 infection was found in 2482, 411 were admitted to hospital with covid-19, and 450 died with covid-19 during the study period. In parallel, 1828 nursing home staff and 2968 healthcare workers were found to have SARS-CoV-2 infection, but fewer than five were admitted or died with covid-19. The adjusted hazard ratio for SARS-CoV-2 infection after two doses of vaccine was 0.09 (95% confidence interval 0.08 to 0.11) for nursing home residents, 0.20 (0.17 to 0.24) for nursing home staff, and 0.13 (0.11 to 0.16) for healthcare workers. Adjusted hazard ratios for hospital admission and mortality after two doses of vaccine were 0.05 (0.04 to 0.07) and 0.03 (0.02 to 0.04), respectively, for nursing home residents. Nursing home staff and healthcare workers recorded insufficient events for mortality analysis.

**Conclusions:**

Vaccination was associated with 80-91% reduction in SARS-CoV-2 infection in all three cohorts and greater reductions in hospital admissions and mortality among nursing home residents for up to five months. More data are needed on longer term effects of covid-19 vaccines.

## Introduction

The global pandemic of covid-19 has caused more than 195 million confirmed cases and 4 million deaths to date.^1^ Spain is one of the most affected countries in Europe, reporting more than 4 million cases by July 2021, the second highest figure among the European Union and European Economic Area member states.^2^

Three vaccines had been approved by the European Medicines Agency at the time of writing—Pfizer-BioNTech BNT162b2 mRNA, Moderna mRNA-1273, and Oxford-AstraZeneca ChAdOx1 nCoV-19.^3^ All three have shown high efficacy in clinical trials, with 95% efficacy against symptomatic covid-19 for BNT162b2 mRNA, 94.1% for mRNA-1273, and 70.4% for ChAdOx1 nCoV-19.^4^ ^5^ ^6^ However, the trials have not included large enough sample sizes to provide reliable evidence of protection against severe disease or mortality.

Certain population groups, such as nursing home residents, have been under-represented in existing clinical trials of covid-19 vaccination, despite evidence suggesting that nursing home residents and staff are disproportionately affected by covid-19. Nursing homes accounted for a large proportion of deaths globally and showed a disproportionately higher mortality than the general population of the same age.^7^ ^8^ ^9^ Preventing outbreaks of infections and reducing related mortality in nursing home settings is crucial for minimising the impact of the pandemic. However, no trial has studied this population specifically. Determining how effective covid-19 vaccines are in these high risk populations is important.

Little is known about the effectiveness of the approved vaccines in actual practice conditions, outside research settings. Differences in compliance with vaccine doses and intervals, testing for SARS-CoV-2 infection, and management of covid-19 in busy clinical settings may all affect the external validity of reported trial data.

Spain started its mass immunisation programme on 27 December 2020, soon after the first covid-19 vaccine (BNT162b2 mRNA) was approved earlier that month. No data have been reported yet on compliance with vaccination and its observable effects in the Spanish population. We aimed to characterise the first three cohorts of vaccinated people (nursing home residents, nursing home staff, and healthcare workers) and estimate the short term effectiveness of the BNT162b2 mRNA vaccine in preventing infections, hospital admissions, and deaths.

## Methods

### Study design and setting

Our prospective cohort study included three populations that were analysed separately: we identified nursing home residents and nursing home staff from primary care records and administrative data, and healthcare workers from a bespoke registry of healthcare workers. Vaccination status against SARS-CoV-2 infection came from the Catalan Shared Clinical Records, a clinical database of electronic medical records that links primary care and hospital diagnoses and treatments for the whole universal Catalan health system. We further linked data to the regional central database of reverse transcriptase polymerase chain reaction (RT-PCR) and lateral flow tests for SARS-CoV-2, hospital admissions, mortality registries, and primary care electronic health records. Ninety per cent of primary care practices in Catalonia, and 90% of the population, were included in the dataset. Information on professional roles for the included healthcare workers came from the workforce census of the Institut Catala de la Salut. Data from these databases have been previously validated and used for epidemiological research,^10^ ^11^ ^12^ including many studies of covid-19.^7^ ^13^ ^14^ ^15^

### Participants and follow-up

We included all people alive in Catalonia at the beginning of the covid-19 vaccination campaign with BNT162b2 mRNA on 27 December 2020 who were nursing home residents or staff eligible for vaccination or who were identified as healthcare workers. We excluded those with a previous SARS-CoV-2 infection identified by a positive RT-PCR or lateral flow test and those who were not assigned to one of the primary care practices contributing to our database.

We followed non-vaccinated participants from the beginning of the vaccination campaign until the earliest of first dose of vaccine plus 14 day run-in (they then switched to the “single dose vaccinated” arm), an outcome (positive RT-PCR or lateral flow test for SARS-CoV-2 or hospital admission, intensive care admission, or death with covid-19) or the end of the study (26 May 2021). We followed participants vaccinated with a single dose from the day they received the first dose of the vaccine plus 14 days of run-in until the earliest of a second dose of vaccine (they then switched to the “two dose vaccinated” arm), an outcome, or the end of the study. We followed two dose vaccinated participants from the day they received their second dose until an outcome or the end of the study. We treated exposure as time varying, with a participant able to contribute person days of follow-up to all three arms. We did an additional analysis comparing time before first dose with the 14 days after the date of the first dose as a measure of residual confounding (see Statistical analysis).

### Outcomes

We studied SARS-CoV-2 infection, hospital admission with covid-19, and covid-19 as cause of death. We defined SARS-CoV-2 infection by the date of the earliest of a positive RT-PCR or lateral flow test, regardless of symptoms. Screening of all nursing home staff and residents using RT-PCR was conducted after any one case was identified. In addition, RT-PCR and lateral flow tests were recommended among healthcare workers on a fortnightly basis and after three or more weeks of absence (for example, after a holiday or leave). We considered hospital admission to be the date of admission for covid-19 as reported in a bespoke official covid-19 inpatient registry. Death due to covid-19 was based on the reported diagnosis in the mortality registry.

### Additional variables and potential confounders

We assessed individual level sociodemographics and clinical features at the time of inclusion, as collected from primary care electronic health records: age (in years), sex, residence status (nursing home resident or staff) or profession (healthcare worker); we assessed pre-existing comorbidities if present any time before the index date; and we identified long term use of medicines on the basis of primary care prescriptions if active/ongoing on the index date. Lists of ICD-10-CM (international classification of diseases, 10th revision, clinical modification) codes for comorbidities and lists of medicines identified using Anatomical Therapeutic Chemical Classification System codes are provided in supplementary table A.

### Statistical analysis

For descriptive analysis, we expressed continuous variables as mean (standard deviation) or median (interquartile range) and summarised categorical variables as number (percentage). We analysed the existence of confounding by indication by using the standardised mean difference of all confounders listed above to compare vaccinated and unvaccinated groups. We considered a standardised mean difference >0.1 to be equivalent to a relevant imbalance and adjusted for it in multivariable analyses.^16^ Additionally, we did an analysis of effects in the first 14 days after the first dose to assess the likely presence of residual confounding due to participant (unrecorded) variables, cluster effects at nursing home level, or changes in epidemiological parameters related to the covid-19 pandemic at the community level. Any departure from the expected null effect (hazard ratio=1) in these first 14 days after first dose vaccination can be interpreted as a measure of residual confounding.

We analysed vaccination as a time varying exposure with three follow-up intervals. (1) No vaccination: from 27 December 2020 until first dose vaccination plus 14 day run-in (where applicable), outcome, or end of study. (2) One dose vaccination: from date of first dose administration plus 14 day run-in to date of second dose, outcome, or end of study. (3) Two dose vaccination: from date of second dose administration to outcome or end of study. For each of these periods, we calculated the rate of outcomes per 10 000 person days by dividing the number of observed events within a period by the number of days of exposure, multiplied by 10 000.

We plotted Kaplan-Meier estimates for each study outcome stratified by vaccination status for visualisation. We fitted random effects time varying Cox models to calculate hazard ratios and 95% confidence intervals for each study outcome according to vaccination status. All Cox models used the index month as a random effect and were adjusted for any confounders with a standardised mean difference >0.1. Three models were conducted separately for each of the cohorts (nursing home residents, nursing home staff, and healthcare workers). We assessed proportionality of hazards in the Cox models by visual inspection of scaled Schoenfeld residuals. We used R version 3.5.1 for all analyses.

### Sensitivity analyses

We did a sensitivity analysis as suggested after peer review, in which we excluded participants who never received a vaccine during the study period. These analyses were therefore entirely focused on the time varying exposure in vaccinated participants, potentially further reducing confounding by indication.

### Patient and public involvement

No patients or members of the public were directly involved in the design or analysis of the reported data. Because of covid-19 related restrictions, interaction with relevant patients, and especially with nursing home residents, has been difficult. Some of the co-authors are healthcare workers and therefore represented in some of our analyses.

## Results

**Fig 1:**
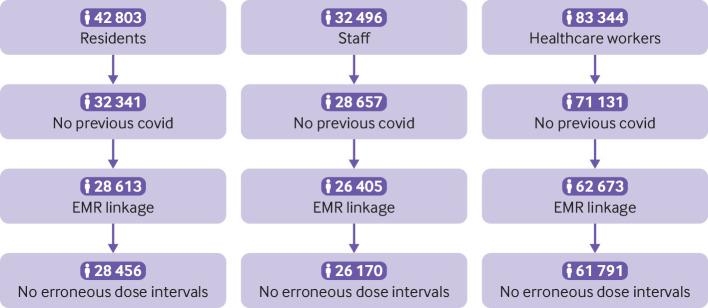
Population flowchart. EMR=electronic medical records

Before exclusions, data for 42 803 nursing home residents, 32 496 nursing home staff, and 83 344 healthcare workers were available for the study. We excluded 10 462 (24.4%) nursing home residents, 3839 (11.8%) nursing home staff, and 12 213 (14.7%) healthcare workers as they had previously been infected with SARS-CoV-2. We also excluded 3885 nursing home residents, 2487 nursing home staff, and 9340 healthcare workers owing to a lack of linked primary care records or dose interval/s or date data (fig 1). We therefore analysed data from 28 456 nursing home residents, 26 170 nursing home staff, and 61 791 healthcare workers. Supplementary table B reports professional roles for the included healthcare workers, and supplementary table C shows sociodemographics and vaccination status for the excluded populations compared with the analysed populations.

**Fig 2:**
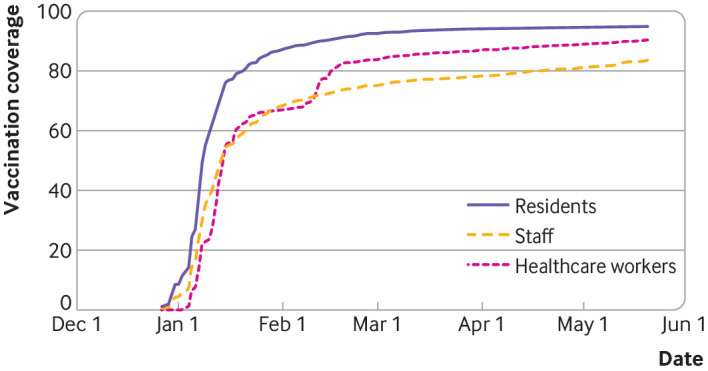
Covid-19 vaccine uptake expressed as percentage with at least one dose (y axis) over time (x axis) in nursing home residents, nursing home staff, and healthcare workers

By the end of the study period, 26 987 (94.8%) nursing home residents, 21 870 (83.6%) nursing home staff, and 55 790 (90.3%) healthcare workers had been vaccinated with at least one dose. Figure 2 shows rapid uptake of vaccine in the three cohorts over the study period, with >50% of participants having received at least one dose by mid-January 2021, and earlier for nursing home residents. Second doses were administered within a median 21 (interquartile range 0) days after the first dose. Supplementary figure A depicts the average weekly incidence of covid-19 in nursing home residents and the general population for context.

Table 1 and supplementary figure B compare the vaccinated and unvaccinated groups of the three cohorts. Few differences existed between vaccinated and unvaccinated nursing home residents. Vaccinated residents had a mean age of 85.7 years and were 73.3% female, whereas unvaccinated residents had a mean age of 85.6 years and were 70.1% female. They had a similar prevalence of comorbidities and use of most medicines except sedatives/hypnotics and antidepressants. All other covariates were sufficiently balanced without adjustment (standardised mean difference ≤0.1). Vaccinated and unvaccinated nursing home staff were also similar in terms of sex, but differed in age, prevalence of some comorbidities (obesity, hypertension, osteoarthritis), and medicine use (lipid modifying drugs and angiotensin converting enzyme inhibitors/angiotensin receptor blockers), all with standardised mean difference >0.1. Vaccinated healthcare workers differed (standardised mean difference >0.1) from unvaccinated ones in terms of age (43.7 *v* 39.9 years), sex (75.2% *v* 79.7% female), prevalence of hypertension (7.8% *v* 5.3%), and history of use of lipid modifying agents (4.6% *v* 2.5%), with no other relevant differences observed.

**Table 1:** Baseline characteristics stratified by vaccination (none versus any) status. Values are numbers (percentages) unless stated otherwise

Characteristics	Nursing home residents			Nursing home staff			Healthcare workers	
	Ever vaccinated (n=26 987)	Never vaccinated (n=1469)		Ever vaccinated (n=21 870)	Never vaccinated (n=4300)		Ever vaccinated (n=55 790)	Never vaccinated (n=6001)
Mean (SD) age, years	85.70 (9.08)	85.58 (11.33)		45.18 (12.6)	39.99 (12.83)		43.71 (12.27)	39.92 (12.73)
Female sex	19 794 (73.3)	1030 (70.1)		19 013 (86.9)	3788 (88.1)		41 952 (75.2)	4783 (79.7)
Analgesics	13 993 (51.9)	757 (51.5)		1667 (7.6)	304 (7.1)		3081 (5.5)	332 (5.5)
Sedatives/hypnotics	10 639 (39.4)	504 (34.3)		2155 (9.9)	362 (8.4)		4555 (8.2)	449 (7.5)
Anticoagulants	11 503 (42.6)	615 (41.9)		385 (1.8)	79 (1.8)		1031 (1.8)	155 (2.6)
Antidepressants	12 983 (48.1)	627 (42.7)		2199 (10.1)	393 (9.1)		4731 (8.5)	467 (7.8)
Antiepileptics	4627 (17.1)	229 (15.6)		651 (3.0)	113 (2.6)		1409 (2.5)	128 (2.1)
Antipsychotics	11 650 (43.2)	596 (40.6)		224 (1.0)	43 (1.0)		409 (0.7)	57 (0.9)
Antacids	13 970 (51.8)	731 (49.8)		1495 (6.8)	213 (5.0)		3471 (6.2)	269 (4.5)
Systemic corticoids	950 (3.5)	77 (5.2)		177 (0.8)	34 (0.8)		451 (0.8)	48 (0.8)
Oral antidiabetics	4003 (14.8)	206 (14.0)		552 (2.5)	70 (1.6)		941 (1.7)	58 (1.0)
Insulin	2260 (8.4)	146 (9.9)		191 (0.9)	30 (0.7)		397 (0.7)	28 (0.5)
Lipid modifying agents	5494 (20.4)	248 (16.9)		1122 (5.1)	117 (2.7)		2582 (4.6)	147 (2.4)
α blockers	308 (1.1)	14 (1.0)		16 (0.1)	5 (0.1)		52 (0.1)	3 (<0.1)
Other antihypertensives	115 (0.4)	7 (0.5)		5 (<0.1)	0		19 (<0.1)	2 (<0.1)
β blockers	4965 (18.4)	292 (19.9)		482 (2.2)	68 (1.6)		1361 (2.4)	93 (1.5)
Calcium channel blockers	3940 (14.6)	204 (13.9)		327 (1.5)	54 (1.3)		692 (1.2)	50 (0.8)
Combination antihypertensives	2403 (8.9)	99 (6.7)		627 (2.9)	83 (1.9)		1086 (1.9)	77 (1.3)
Diuretics	8707 (32.3)	507 (34.5)		410 (1.9)	44 (1.0)		775 (1.4)	67 (1.1)
ACE inhibitors/ARBs	7860 (29.1)	405 (27.6)		1119 (5.1)	131 (3.0)		2393 (4.3)	156 (2.6)
Chronic obstructive pulmonary disease/asthma inhalers	3465 (12.8)	201 (13.7)		862 (3.9)	150 (3.5)		2262 (4.1)	241 (4.0)
Atrial fibrillation	4491 (16.6)	286 (19.5)		56 (0.3)	7 (0.2)		224 (0.40)	14 (0.2)
Osteoarthritis	11 636 (43.1)	584 (39.8)		1833 (8.4)	252 (5.9)		2452 (4.4)	228 (3.8)
Asthma	1228 (4.6)	65 (4.4)		1185 (5.4)	229 (5.3)		3467 (6.2)	366 (6.1)
Ischaemic heart disease	2313 (8.6)	116 (7.9)		89 (0.4)	13 (0.3)		257 (0.5)	19 (0.3)
Dementia	11 175 (41.4)	613 (41.7)		4 (<0.1)	0		3 (<0.1)	0
Diabetes mellitus	7053 (26.1)	392 (26.7)		710 (3.2)	93 (2.2)		1204 (2.2)	91 (1.5)
Liver disease	1005 (3.7)	54 (3.7)		665 (3.0)	93 (2.2)		1042 (1.9)	96 (1.6)
Hypertension	18 454 (68.4)	1002 (68.1)		2405 (11.0)	287 (6.7)		4368 (7.8)	318 (5.3)
Heart failure	3137 (11.6)	189 (12.9)		20 (0.1)	2 (<0.1)		34 (0.1)	5 (0.1)
Cerebrovascular disease	2911 (10.8)	187 (12.7)		92 (0.4)	16 (0.4)		177 (0.3)	12 (0.2)
Chronic obstructive pulmonary disease	2249 (8.3)	125 (8.5)		169 (0.8)	30 (0.7)		269 (0.5)	23 (0.4)
Chronic kidney disease	6894 (25.6)	404 (27.5)		124 (0.6)	12 (0.3)		282 (0.5)	18 (0.3)
Cancer (all except non-melanoma skin cancer)	4864 (18.0)	278 (18.9)		616 (2.8)	84 (2.0)		2080 (3.7)	188 (3.1)
Obesity	5493 (20.4)	253 (17.2)		3894 (17.8)	595 (13.8)		5002 (9.0)	480 (8.0)
Valvular disease	1651 (6.1)	100 (6.8)		128 (0.6)	24 (0.6)		383 (0.7)	40 (0.7)
Hepatitis B	81 (0.3)	7 (0.5)		54 (0.2)	21 (0.5)		61 (0.1)	5 (0.1)
Hepatitis C	305 (1.1)	20 (1.4)		85 (0.4)	12 (0.3)		99 (0.2)	11 (0.2)
HIV infection	14 (0.1)	1 (0.1)		72 (0.3)	7 (0.2)		69 (0.1)	7 (0.1)

ACE=angiotensin converting enzyme; ARB=angiotensin receptor blocker.

Supplementary figure C depicts the number of participants tested over time. By the end of the study period, a total of 25 834 (95.7%) vaccinated and 1223 (83.3%) unvaccinated nursing home residents, 21 185 (96.9%) vaccinated and 3589 (83.5%) unvaccinated nursing home staff, and 35 699 (64.0%) vaccinated and 4158 (69.3%) unvaccinated healthcare workers had been tested at least once. A median of 3 (interquartile range 1-5) tests were performed during the study period in vaccinated and 3 (2-5) in unvaccinated nursing home residents, 11 (8-16) and 9 (4-14) in staff, and 3 (1-5) and 3 (2-5) among healthcare workers.

**Fig 3:**
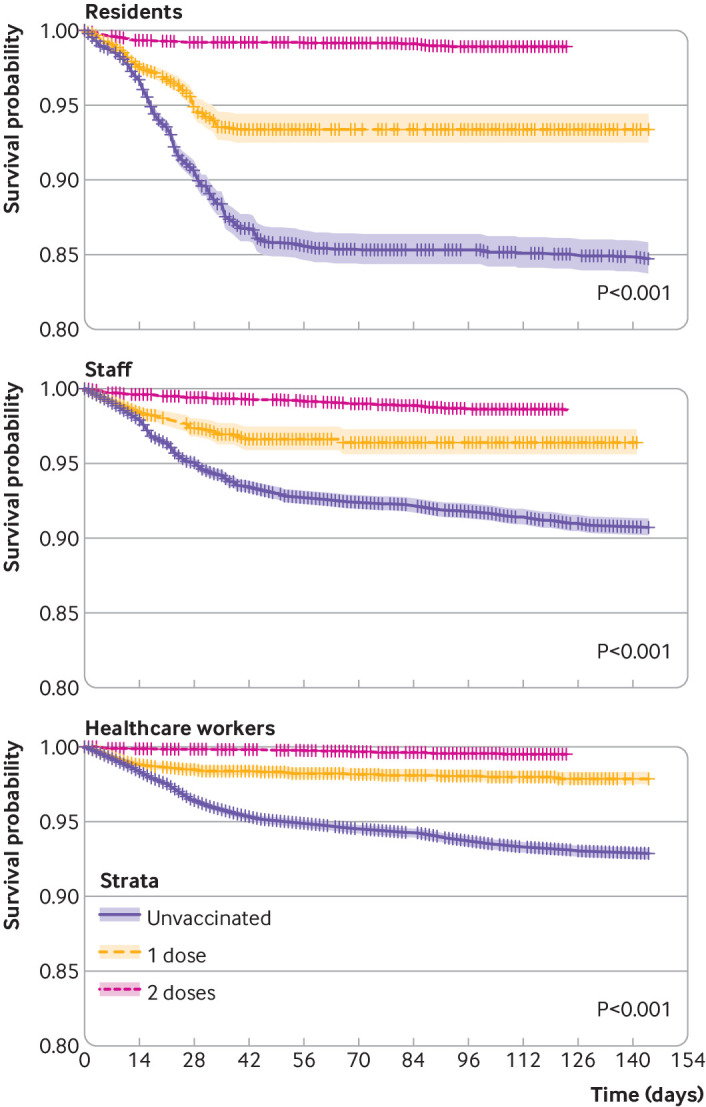
Kaplan-Meier estimates of SARS-CoV-2 infection according to vaccination status in nursing home residents (top left), staff (bottom left), and healthcare workers (top right)

In total, 1335 SARS-CoV-2 infections occurred among unvaccinated nursing home residents, 620 in the first 14 days following the first dose, and 1147 among vaccinated residents. Most post-vaccination infections were after the first vaccine dose (882), with few after the second dose (265). The incidence rates of infection were 22.6/10 000 person days for unvaccinated residents, 14.3/10 000 for residents with one dose, and 1.0/10 000 for residents with two doses. Any vaccination led to an adjusted hazard ratio for SARS-CoV-2 infection of 0.21 (95% confidence interval 0.19 to 0.24). A single vaccine dose led to an adjusted hazard ratio of 0.53 (0.49 to 0.58) and a second dose to an adjusted hazard ratio of 0.09 (0.08 to 0.11) (table 2). Figure 3 shows Kaplan-Meier plots stratified by vaccination status, in which a modest but noticeable reduction in infections was apparent earlier than expected, already seen in the first 14 days among the vaccinated versus unvaccinated residents. The resulting adjusted hazard ratio of 0.77 (0.69 to 0.86) is a measure of residual confounding.

**Table 2:** Number, incidence rates, and adjusted hazard ratios for covid-19 according to vaccination status in nursing home residents, nursing home staff, and healthcare workers

Cohort and period	**Population**	**Cases**	**Exposure person days**	**Exposure days (mean)**	**Rate per 10 000 person days**	**Adjusted hazard ratio (95% CI)**
**Nursing home residents**						
Unvaccinated	28 456	1335	590 956	20.77	22.59	Reference
Days 0-14 after first dose	26 044	620	360 880	13.86	17.18	0.77 (0.69 to 0.86)
Vaccinated (from day 14 after first dose)	25 375	527	2 841 387	111.98	1.86	0.21 (0.19 to 0.24)
Vaccinated—one dose, from day 14	26 044	882	616 788	23.68	14.3	0.53 (0.49 to 0.58)
Vaccinated—two doses, from second dose date	24 484	265	2 585 479	105.6	1.03	0.09 (0.08 to 0.11)
**Nursing home staff**						
Unvaccinated	26 170	1,144	1 121 942	42.87	10.19	Reference
Days 0-14 after first dose	21 269	338	291 473	13.70	11.6	0.89 (0.71 to 1.02)
Vaccinated (from day 14 after first dose)	20 429	346	2 151 971	105.34	1.61	0.22 (0.19 to 0.24)
Vaccinated—one dose, from day 14	21 269	433	490 062	23.04	8.84	0.62 (0.55 to 0.69)
Vaccinated—two doses, from second dose date	19 513	251	1 953 382	100.11	1.28	0.20 (0.17 to 0.24)
**Healthcare workers**						
Unvaccinated	61 791	1961	2 269 003	36.72	8.64	Reference
Days 0-14 after first dose	54 848	649	758 224	13.82	8.56	0.97 (0.87 to 1.08)
Vaccinated (from day 14 after first dose)	53 585	358	5 528 745	103.18	0.65	0.13 (0.11 to 0.14)
Vaccinated—one dose, from day 14	54 848	785	1 409 807	25.70	5.57	0.60 (0.55 to 0.66)
Vaccinated—two doses, from second dose date	51 019	222	4 877 162	95.6	0.46	0.13 (0.11 to 0.16)

We found similar results for nursing home staff, with 1144 infections among unvaccinated staff and 684 among vaccinated staff. Most infections among vaccinated staff were after the first vaccine dose (433), not the second (251). The incidence rates of infection were 10.2/10 000 person days for unvaccinated staff, 8.8 for staff with one dose, and 1.3 for staff with two doses. Any vaccination led to an adjusted hazard ratio for infection of 0.22 (0.19 to 0.24). A single vaccine dose led to an adjusted hazard ratio of 0.62 (0.55 to 0.69), and a second dose led to an adjusted hazard ratio of 0.20 (0.17 to 0.24) (table 2; fig 3). The adjusted hazard ratio associated with one dose of vaccine during the first 14 days was 0.89 (0.71 to 1.02), suggesting the absence of relevant unresolved confounding.

Finally, in the cohort of healthcare workers, 1961 unvaccinated and 1007 vaccinated staff tested positive for SARS-CoV-2 (785 after one dose, 222 after the second). The incidence rates of infection were 8.6/10 000 person days among unvaccinated healthcare workers, 5.6/10 000 after one dose, and 0.5/10 000 after two doses. Any vaccination led to an adjusted hazard ratio of 0.13 (0.11 to 0.14). A single dose led to an adjusted hazard ratio of 0.60 (0.55 to 0.66), and a second dose led to an adjusted hazard ratio of 0.13 (0.11 to 0.16) (table 2; fig 3). The adjusted hazard ratio for the first 14 days after first dose vaccination was 0.97 (0.87 to 1.08), suggesting the absence of residual confounding.

A sensitivity analysis excluding the “never vaccinated” nursing home residents resulted in a hazard ratio of 0.98 (0.87 to 1.10), suggesting further reductions in confounding in these analyses. The corresponding adjusted hazard after a second dose of vaccine was 0.11 (0.09 to 0.13). More detail is reported in supplementary table D.

**Fig 4:**
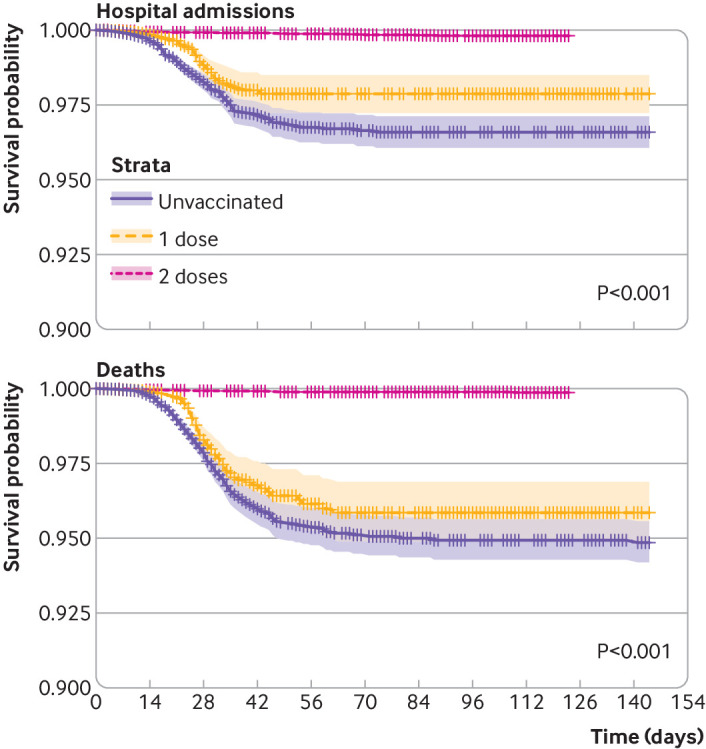
Kaplan-Meier estimates of hospital admission (left) and mortality with covid-19 (right) according to vaccination status in nursing home residents

Hospital admissions with covid-19 were recorded for 411 nursing home residents, with incidence rates of 3.6/10 000 person days for unvaccinated residents, 2.1/10 000 after one dose, and 0.2/10 000 after two doses. Any vaccination led to an adjusted hazard for admission of 0.35 (0.28 to 0.43). One vaccine dose led to an adjusted hazard ratio of 0.43 (0.34 to 0.54), and a second dose led to a hazard ratio of 0.05 (0.04 to 0.07) (table 3). Figure 4 shows Kaplan-Meier plots for hospital admissions in nursing home residents by vaccination status.

**Table 3:** Number of hospital admissions and deaths, incidence rates, and adjusted hazard ratios according to vaccination status among nursing home residents

Cohort and period	**Population**	**Cases**	**Exposure person days**	**Exposure days (mean)**	**Rate per 10 000 person days**	**Adjusted hazard ratio (95% CI)**
**Hospital admission**						
Unvaccinated	28 456	228	631 699	22.20	3.61	Reference
Vaccinated (from day 14 after first dose)	26 780	143	3 009 349	112.37	0.48	0.35 (0.28 to 0.43)
Vaccinated—one dose, from day 14	26 887	134	655 958	24.40	2.05	0.43 (0.34 to 0.54)
Vaccinated—two doses, from second dose date	25 945	49	2 729 304	105.20	0.18	0.05 (0.04 to 0.07)
**Death**						
Unvaccinated	28 456	272	639 181	22.46	4.26	Reference
Vaccinated (from day 14 after first dose)	26 927	153	3 030 779	112.56	0.51	0.31 (0.26 to 0.39)
Vaccinated—one dose, from day 14	27 000	145	662 666	24.54	2.19	0.49 (0.39 to 0.61)
Vaccinated—two doses, from second dose date	26 126	33	2 745 713	105.10	0.12	0.03 (0.02 to 0.04)

We observed 450 deaths among nursing home residents: 272 before vaccination, 145 after one dose of vaccine, and 33 after the second dose. Incidence rates of covid-19 mortality were 4.3/10 000 person days in unvaccinated residents and 0.5/10 000 in all vaccinated residents (2.2/10 000 after one dose and 0.1/10 000 after two doses). Any vaccination led to an adjusted hazard ratio for death with covid-19 of 0.31 (0.26 to 0.39). One vaccine dose led to an adjusted hazard ratio of 0.49 (0.39 to 0.61), and two doses led to a hazard ratio of 0.03 (0.02 to 0.04) (table 3). Figure 4 shows Kaplan-Meier plots for mortality in nursing home residents by vaccination status.

We recorded hospital admissions for 29 (27 unvaccinated) nursing home staff and 64 (48 unvaccinated) healthcare workers and 0 and <5 deaths respectively. We did not model these owing to limited statistical power.

## Discussion

This is the first report of the clinical effectiveness of a covid-19 vaccine in nursing homes globally to our knowledge, and the first set of results on the effect of vaccination to prevent covid-19 in Spain. Using a comprehensive linked database that combined primary care, screening and diagnostic RT-PCR and lateral flow test, hospital, and mortality data, we studied the effects of the BNT162b2 mRNA vaccine in the three populations prioritised for vaccination nationally. Partial (single dose) vaccination resulted in 40-50% reductions in SARS-CoV-2 infections, whereas full (two dose) vaccination led to 80-90% protection. The observed effects in nursing home residents were similar to those seen in healthcare workers and nursing home staff, and in line with previous phase III trials.

We also found significant, clinically relevant reductions in the risks of severe covid-19 among nursing home residents, amounting to a striking 95% and 97% reduction in hospital admission and mortality risks, respectively, after two doses of BNT162b2. A plain English summary of the observed results is reported in supplementary table E.

### Findings in context

Ambitious covid-19 vaccination campaigns are ongoing around the world, but knowledge is still scarce on the real world effectiveness of covid-19 vaccines, especially in high risk populations such as nursing home residents and healthcare workers. Although vaccines have shown high efficacy in clinical trials, real world evidence on effectiveness is needed to confirm their effects in routine practice settings and among populations under-represented in pivotal trials. A recent study with national population level Scottish data showed that by the fourth week after the first dose, the BNT162b2 and ChAdOx1 vaccines reduced the risk of hospital admission by 85% (95% confidence interval 76% to 91%) and 94% (73% to 99%), respectively. It also showed that two doses of the BNT162b2 mRNA vaccine offered more than 85% protection against symptomatic infection among people aged over 80.^17^ A prospective cohort study of hospital staff in England reported vaccine effectiveness of 72% (58% to 86%) in the 21 days after the first dose of the BNT162b2 mRNA vaccine.^18^ A cohort study of healthcare workers in Israel reported a 75% reduction in SARS-CoV-2 infection and 85% (71% to 92%) reduction in symptomatic covid-19 during the 15-28 days after the first dose of BNT162b2.^19^ A recent study including more than half a million people in Israel estimated the effectiveness of the BNT162b2 vaccine against infection at 46% (40% to 51%) 14-20 days after the first dose and 92% (88% to 95%) one week after the second dose.^20^ Interim results have been published on the effectiveness of covid-19 vaccines in nursing homes in the United States, with findings consistent with ours.

We found that one dose of the BNT162b2 vaccine reduced the risk of infection by 47% in nursing home residents, 38% in nursing home staff, and 40% in healthcare workers. These results are equivalent to the reported vaccine efficacy of 52.4% between the first and second doses in the phase III trial of BNT162b2 mRNA.^5^ In line with phase III trial findings, our results highlight that vaccine recipients should be told about the modest protection seen between the first and second doses and encouraged to continue shielding, physical distancing, and other protective measures, especially during the first two weeks after the initial dose.

Our study differs from the trial in setting, participants, and outcome ascertainment. Although the trial included only symptomatic covid-19, we included any RT-PCR or lateral flow test positive infection, including regular screening testing. The three populations included were screened periodically during the study period to minimise potential outbreaks: whereas nursing homes conducted universal RT-PCR among staff and residents every time a case was identified, healthcare workers were tested (with RT-PCR or lateral flow test) every fortnight and after a leave of absence of more than three weeks. The observed reduction in infection rates including screening tests is of high relevance and supports recent data suggesting that vaccination could reduce overall transmission of the virus.^18^

### Limitations and strengths of study

Our study has several limitations. The observational nature of our data may have led to confounding by indication. Comprehensive linkage to primary care and hospital records allowed us to measure differences in sociodemographics, comorbidity, and medication usage. Despite this, we identified mild (approximately 20%) reductions in SARS-CoV-2 infections among healthcare workers during the first 14 days after the first dose, suggesting unobserved confounding or nursing home protective effects. Our sensitivity analysis excluding the “never vaccinated” participants reduced confounding further and showed the expected null effect in the 14 days after the first dose of vaccine, in line with phase III trials. The findings from these analyses are in line with the primary analyses.

Changes in community transmission over the study period are depicted in supplementary figure A, which shows an improved overall situation in community transmission from the second half of February 2021, with further improvements in nursing homes. Despite a further increase in the form of a minor wave in community transmission after Easter (April 2021), we observed no evidence of this among nursing home residents. In addition, cautious behaviour and increased personal protection during the first two weeks after vaccination could contribute to the unexpected reduction in infection risk observed during that period. Such measures were not recommended in pivotal trials as participants were blinded to vaccine exposure and neither participants nor investigators had knowledge of the lack of effect during the first 12 days after vaccination. Finally, we observed differences in testing practice and management of visits to nursing homes over time. Testing practices changed over time during the study period, particularly in nursing homes. Screening of nursing home residents and staff was reinforced in January 2021 but focused on unvaccinated staff from mid-February, as shown in supplementary figure C. Similarly, guidelines on visits to nursing homes and on temporary exits from them changed over the study period. Visits to nursing homes were minimised in January when risk of covid-19 was high, and exits were not allowed unless they were programmed and for a duration of three or more days. These measures were relaxed and became more flexible when the situation improved from the second half of February, and further in mid-May. Incorporating index month in our random effects model accounted for this. However, higher testing rates in the vaccinated population could have resulted in a higher likelihood of diagnosis. The resulting surveillance bias would result in an underestimation of vaccine effectiveness.

This study also has strengths. The comprehensive linkage and coverage in our database is unique, including primary care, hospital, RT-PCR and lateral flow test results, and mortality data for more than 90% of the regional population. The Catalan health system is universal, minimising dropouts and maximising the completeness of outcome ascertainment. Access to basic sociodemographics and events for people excluded from the analysis allowed us to measure potential selection bias. Our included study population allowed us to study the effects of vaccination against hospital admission and death in nursing home residents, a population subgroup extremely vulnerable to severe and lethal forms of covid-19 and under-represented in previous studies.^7^ ^20^ The pivotal trial was underpowered to analyse these outcomes.^5^

### Conclusions

Our data confirmed that BNT162b2 vaccination strongly reduced the risk of SARS-CoV-2 infection in nursing homes and in healthcare workers, with comparable results to those observed in US based phase III trials and other international observational studies. Hospital admission and death with covid-19 were similarly reduced among nursing home residents, who accounted for a large proportion of deaths with covid-19 in 2020. Although further data and studies are needed to assess the long term effectiveness and safety of this and other covid-19 vaccines, these findings should reassure the population of the major benefits associated with the ongoing vaccination campaign in Spain and elsewhere. Further research is needed to increase our understanding of the effect of vaccination on the management of nursing homes, including visitors, staff, use of protective equipment, and residents themselves.

## What is already known on this topic

Four covid-19 vaccines have been approved for use in the UK and EU to date
A large US based trial found that the first vaccine to be approved, the BNT162b2 mRNA vaccine, had >90% efficacy against symptomatic covid-19
Emerging evidence from observational studies have confirmed similar results in the UK and Israel

## What this study adds

Two dose BNT162b2 vaccination was associated with 80-91% reductions in symptomatic and asymptomatic SARS-CoV-2 infections among nursing home residents, nursing home staff, and healthcare workers
Additionally, vaccination with two doses of BNT162b2 led to ≥95% reductions in covid-19 related hospital admission and mortality among nursing home residents
The effects of two dose vaccination with BNT162b2 in nursing home residents are equivalent to those shown in randomised controlled trial participants

## Data Availability

No patient level data can be shared owing to local information governance and data protection regulations. Aggregated data are available and reported in the supplement.

## Web extra

Extra material supplied by authors

Web appendix: Supplementary materials

## Notes

The lead authors affirm that this manuscript is an honest, accurate, and transparent account of the study being reported; that no important aspects of the study have been omitted; and that any discrepancies from the study as planned (and, if relevant, registered) have been explained.

Dissemination to participants and related patient and public communities: The study results will be disseminated to the public through our media channels, including both national (Spain, the UK) and international media. We have provided a plain English summary table of our findings as supplementary table E.

Provenance and peer review: Not commissioned; externally peer reviewed.
