# Supplementary material for: Associations of BNT162b2 vaccination with SARS-CoV-2 infection and hospital admission and death with covid-19 in nursing homes and healthcare workers in Catalonia: prospective cohort study
Source: BMJ. 2021 Aug 18;374:n1868. doi: 10.1136/bmj.n1868 (PMC8371258; doi:10.1136/bmj.n1868)

SUPPLEMENTARY TABLES AND FIGURES

Supplementary Table A. Co-morbidities and long-term medications included in the analysis.

**Comorbidity**	**ICD10-CM codes**
Asthma	J45, J45.20, J45.21, J45.22
Cancer (except non-melanoma skin cancer)	C00-C97 (except C44), D00-D09
Atrial fibrillation	I48.0. I48.1, I48.2, I48.91
Cerebrovascular disease	G45.0, G45.1, G45.2, G45.8, G45.9, G46.0, G46.1, G46.2, G46.3, G46.4, G46.5, G46.6, G46.7, G46.9, I63.00, I63.10, I63.20, I63.30, I63.40, I63.50, I63.6, I63.8, I63.9, I67.82, I67.9, I69.30, I69.320 I69.398, I69.80,I69.998<br>I61.0, I61.1, I61.2, I61.3, I61.4, I61.5, I61.6, I61.8, I61.9, I61.10
Chronic Kidney Disease	E66.01, E66.09, E66.1, E66.2, E66.8, E66.9
Chronic obstructive pulmonary disease	J43.0, J43.1, J43.2, J43.8, J43.9, J44, J44.0,J44.1,J44.9
Diabetes Mellitus (type I and II)	E10.10, E10.29, E10.311, E10.359, E10.39, E10.49, E10.59, E10.621, E10.65, E10.69, E10.8, E10.9<br>E11.01, E11.21, E11.22, E11.29, E11.311, E11.39, E11.40, E11.43, E11.49, E11.51, E11.59, E11.610. E11.621, E11.638, E11.641, E11.649, E11.65, E11.69, E11.8, E11.9, E13.10, E13.29, E13.39, E13.49, E13.59, E13.641, E13.69, E13.8, E13.9
Heart failure	I50.1, I50.20, I50.30, I50.32, I50.9
HIV infection	Z21,B20, B97.35
Hypertension	I10, I11.0, I11.9, I12.0, I12.9, I13.0, I13.10, I13.2, I15.0,I15.1,I15.2, I15.8,I15.9
Ischaemic heart disease	I20, I20.0, I20.8, I20.9, I24.0, I24.1, I24.8, I24.9, I25, I25.10, I25.2, I25.41, 125.5, I25.6, I25.89, I25.9, I70.90, I21, I21.0, I21.01, I21.02,I21.09, I21.1, I21.11, I21.19, I21.2, I21.29, I21.3, I21.4, I22.0, I22.1, I22.2, I22.8, I22.9, I23.0, I23.1, I23.2, I23.3, I23.4, I23.5, I23.6, I23.8
Liver failure	K70.0, K70.10, K70.2, K70.30, K70.9, K73.0, K73.1, K73.2, K73.8, K73.9, K75.0, K75.2, K75.3, K75.4, K75.89, k75.9, K76.5, K76.7, K76.9
Obesity	E66.01, E66.09, E66.1, E66.2, E66.8, E66.9
Type B Hepatitis	B16.0, B16.1, B16.2, B16.9
Type C Hepatitis	B17.10, B18.2
**Medicine or drug class**	**ATC codes**

Analgesics	M01AX%, N02AA%, N02AB%, N02AC%,N02AD%, N02AE%, N02AX%, N02BA%, N02BB%, N02BE%<br><br>M01AA%, M01AB%, M01AC%, M01AE%, M01AG%, M01AH%.
Sedatives/hypnotics	N05BA%, N05BB%, N05BE%, N05BX%, N05CD%, N05CF%, N05CM%,
Antitcoagulants	B01AA%, B01AB%, B01AC%, B01AE%, B01AF%, B01AX%
Antidepressants	N06AA%, N06AB%, N06AG%, N06AX%
Antieplileptics	N03AA%, N03AB%, N03AC%, N03AD%, N03AE%, N03AF%, N03AG%, N03AX%
Anti-psychotics	N05AA%, N05AB%, N05AC%, N05AD%, N05AE%, N05AF%, N05AH%, N05AX%, N05AK%, N05AL%
Antiacids	A02AB%, A02AD%, A02AX%, A02BA%, A02BB%, A02BC%, A02BX%,
Systemic corticoids	H02AB%, H02BX91
Oral antidiabetics agents	A10BA%, A10BB%, A10BD%, A10BF%, A10BG%,A10BH%, A10BX%, A10BK%, A10BJ%
Insulin	A10AB%, A10AC%,A10AD%, A10AE%
Lipid modifying agents	C10AA%<br>C10AB%, C10AC%, C10AD%, C10AX%, C10BA%, C10BX%,
Alpha blockers	C02CA%
Beta blockers	C07AA%, C07AB%, C07AG%
Calcium channel blockers	C08CA%, C08DA%, C08DB%
Diuretics	C03AA%, C03BA%, C03DA%, C03DB%, C03EA%, C03EB%
ACEI/ARBs	C09AA%, C09CA%
Inhalers	R03AA%, R03AC%, R03AK%, R03AL%, R03BA%, R03BB%

Supplementary Table B. Number (%) of healthcare professionals classified as senior physicians/consultants, nurses, technicians, and administrative/support staff. NOTE: professional role is available for the 67% healthcare workers working for the main healthcare provider in Catalonia (Institut Catala de la Salut)

**Job/Role**	**N**	**%**
Senior physician/consultant	7,453	12.06%
Junior/trainee doctor	1,306	2.11%
Nurse	11,995	19.41%
Specialized middle grade technician	5,406	8.75%
Specialized high grade technician	1,448	2.34%
Administrative services	11,482	18.58%
Other	2,574	4.17%
No information available	20,127	32.57%
**Total**	**61,791**	**100.00%**

Supplementary Table C. Differences in socio-demographics and vaccination status between included and excluded study participants

		**Residents**	**Staff**	**Healthcare workers**
**Excluded**	**Vaccinated**	2,378 (64.31%)	1,698 (83.44%)	5,439 (90.27%)
	**Age**	87 (8.65)	45 (15.09)	43 (12.39)
	**Gender (female)**	2,637 (71.31%)	1,713 (84.18%)	4,341 (72.05%)
**Included**	**Vaccinated**	26,987 (94.84%)	21,870 (83.57%)	55,790 (90.29)
	**Age**	86 (9.21)	44 (12.78)	43 (12.36)
	**Gender (female)**	20,824 (73.18%)	22,801 (87.13%)	46,735 (75.63%)

Supplementary Table D. Number, incidence rates, and adjusted hazard ratios (HR) for COVID-19 according to vaccination status in nursing home residents, nursing home staff, and healthcare workers, after the exclusion of the ‘never vaccinated’ populations

Cohort	Period	Population	Cases	Exposure person-days (N)	Exposure days (mean)	Rate per 10 000 pers-days	Adjusted HR (95% CI)
**Residents**	Unvaccinated person-time	26,987	957	431,849	16.0	22.16	REF
**Residents**	Days 0-14 after first dose	26,030	620	360,684	13.9	17.19	0.98 (0.87 to 1.10)
**Residents**	Vaccinated (from day 14 after 1st dose)	25,361	527	2,840,003	112.0	1.86	0.24 (0.22 to 0.28)
**Residents**	Vaccinated One dose, from day 14	26,030	882	615,208	23.6	14.34	0.65 (0.59 to 0,71)
**Residents**	Vaccinated Two doses, from 2nd dose date	24,484	265	2,585,479	105.6	1.03	0.11 (0.09 to 0.13)
**Staff**	Unvaccinated person-time	21,870	629	561,233	25.7	11.21	REF
**Staff**	Days 0-14 after first dose	21,241	338	291,090	13.7	11.61	1.01 (0.87 to 1.18)
**Staff**	Vaccinated (from day 14 after 1st dose)	20,403	346	2,149,641	105.4	1.61	0.26 (0.23 to 0.30)
**Staff**	Vaccinated One dose, from day 14	21,241	433	487,349	22.9	8.88	0.74 (0.65 to 0.84)
**Staff**	Vaccinated Two doses, from 2nd dose date	19,513	251	1,953,382	100.1	1.29	0.24 (0.20 to 0.28)
**Healthcare workers**	Unvaccinated person-time	55,790	980	1,519,817	27.2	6.45	REF
**Healthcare workers**	Days 0-14 after first dose	54,810	649	757,712	13.8	8.57	1.38 (1.23 – 1.55)
**Healthcare workers**	Vaccinated (from day 14 after 1st dose)	53,551	358	5,526,133	103.2	0.65	0.18 (0.16 – 0.20)
**Healthcare workers**	Vaccinated One dose, from day 14	54,810	785	1,406,683	25.7	5.58	0.93 (0.85 – 1.03)
**Healthcare workers**	Vaccinated Two doses, from 2nd dose date	51,019	222	4,877,162	95.6	0.46	0.22 (0.18 – 0.27)

Supplementary Table E. Plain English summary of observed associations

Population/Group	Health outcome	Estimated effect
Nursing home residents	COVID-19 infection	79% reduction in risk overall<br>47% reduction 14 days after 1st dose<br>91% reduction after 2nd dose
	COVID-19 related hospital admission	65% reduction in risk overall<br>57% reduction 14 days after 1st dose<br>95% reduction after 2nd dose
	COVID-19 related mortality	69% reduction in risk overall<br>51% reduction 14 days after 1st dose<br>97% reduction after 2nd dose
Nursing home staff	COVID-19 infection	78% reduction in risk overall<br>38% reduction 14 days after 1st dose<br>80% reduction after 2nd dose
Healthcare workers	COVID-19 infection	87% reduction in risk overall<br>40% reduction 14 days after 1st dose<br>87% reduction after 2nd dose

Supplementary Figure A. Average weekly incidence of COVID-19 in nursing home residents (top) and in the general population of Catalonia (bottom) in the study period

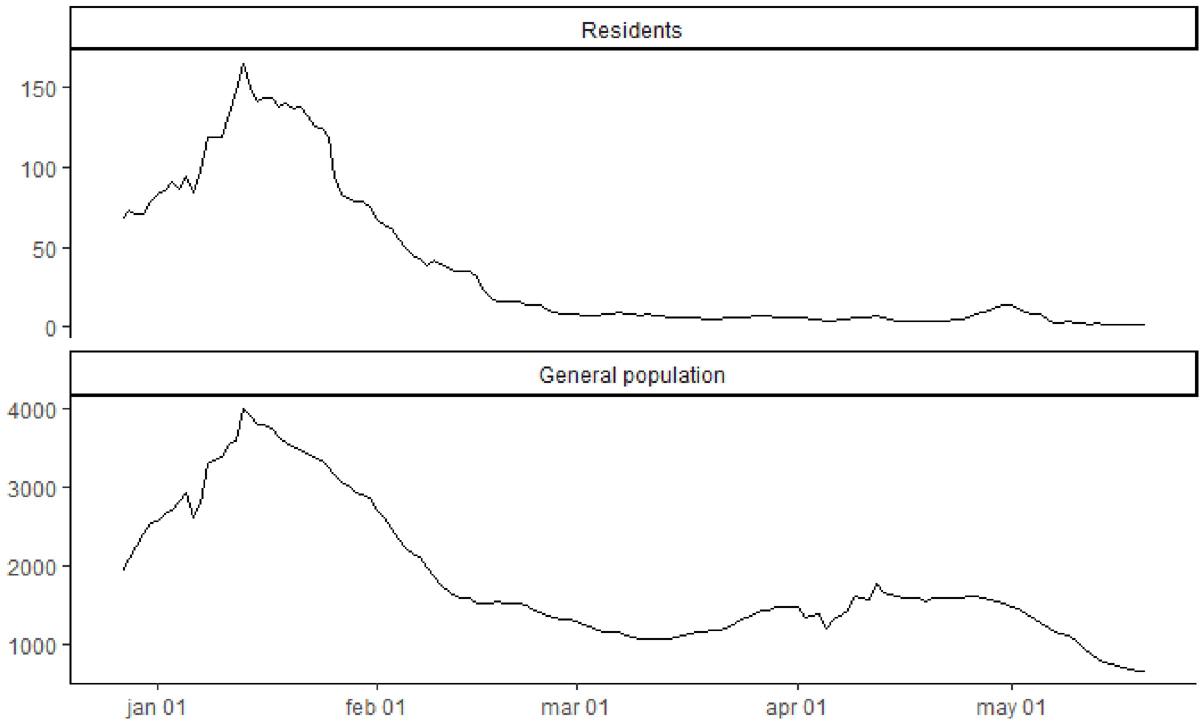

Supplementary Figure B. Standardized mean differences (SMD) in covariate imbalance between the vaccinated (with at least 1 dose) and unvaccinated. Nursing home residents (left), staff (middle) and healthcare workers (right)

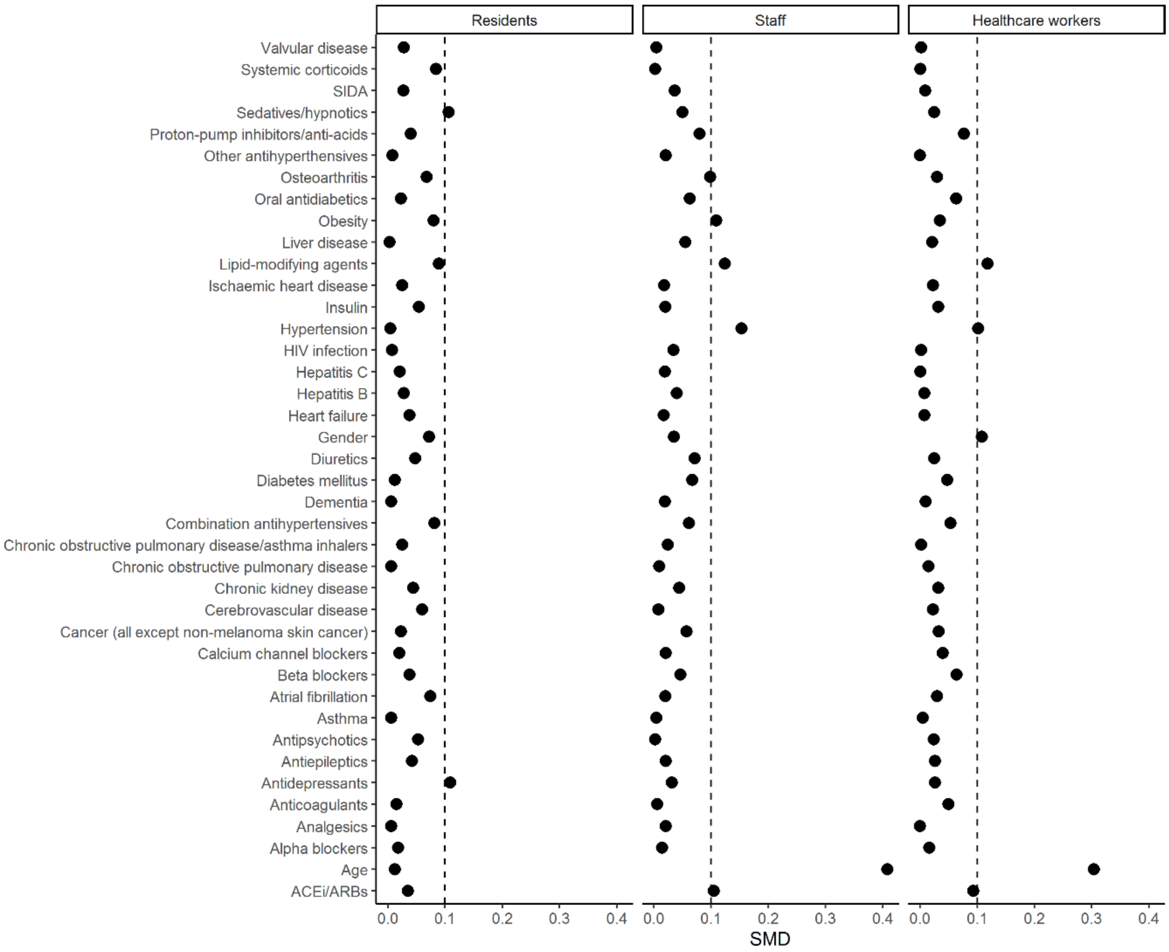

Supplementary Figure C. Incidence rates of PCR testing in nursing home residents (top), staff (middle) and healthcare workers (bottom) in the vaccinated (left) and unvaccinated (right)

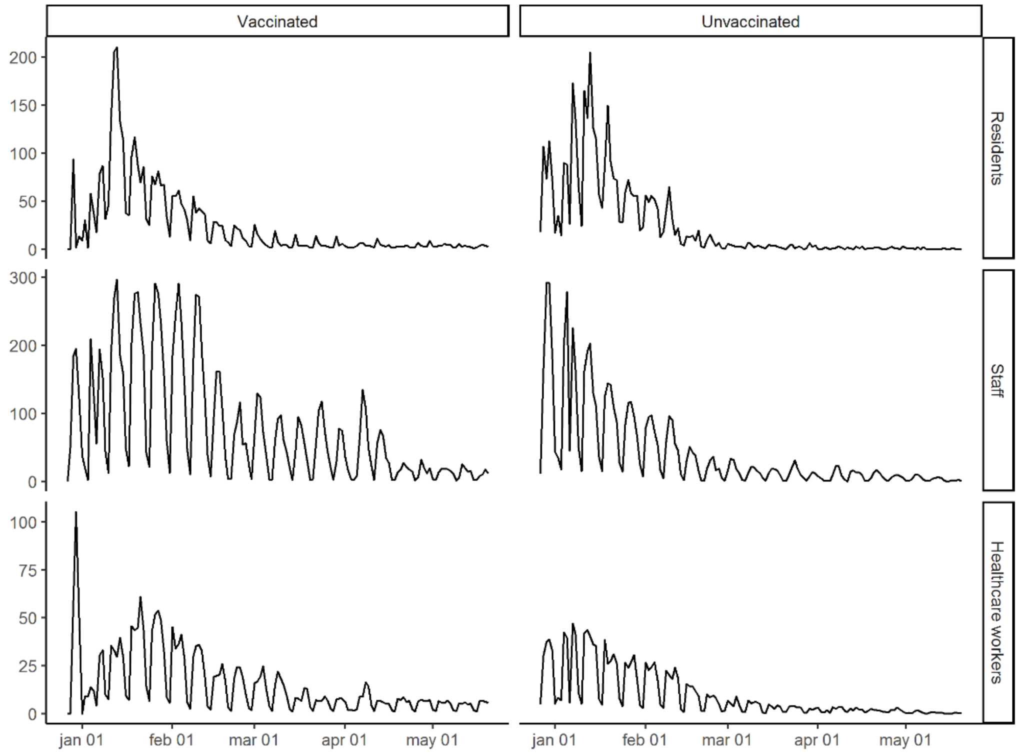

Supplement: Supplementary file 1 — Web appendix: Supplementary materials [file cabc065895.ww1.pdf]
